# A Diagnostic Algorithm To Investigate Pyrazinamide and Ethambutol Resistance in Rifampin-Resistant Mycobacterium tuberculosis Isolates in a Low-Incidence Setting

**DOI:** 10.1128/AAC.01798-18

**Published:** 2019-01-29

**Authors:** Söenke Andres, Matthias I. Gröschel, Doris Hillemann, Matthias Merker, Stefan Niemann, Katharina Kranzer

**Affiliations:** a National Mycobacterium Reference Laboratory, Research Center Borstel, Borstel, Germany; b Molecular and Experimental Mycobacteriology, Priority Research Area Infections, Research Center Borstel, Borstel, Germany; c German Center for Infection Research, Partner Site Borstel, Borstel, Germany; d London School of Hygiene & Tropical Medicine, London, United Kingdom

**Keywords:** *Mycobacterium tuberculosis*, ethambutol, pyrazinamide

## Abstract

Phenotypic drug susceptibility testing (DST) for the two first-line tuberculosis drugs ethambutol and pyrazinamide is known to yield unreliable and inaccurate results. In this prospective study, we propose a diagnostic algorithm combining phenotypic DST with Sanger sequencing to inform clinical decision-making for drug-resistant Mycobacterium tuberculosis complex isolates. Sequencing results were validated using whole-genome sequencing (WGS) of the isolates. Resistance-conferring mutations obtained by *pncA* sequencing correlated well with phenotypic DST results for pyrazinamide. Phenotypic resistance to ethambutol was only partly explained by mutations in the *embB* 306 codon. Additional resistance-conferring mutations were found in the *embB* gene at codons 354, 406, and 497. In several isolates that tested ethambutol susceptibility by phenotypic DST, well-known resistance-conferring *embB* mutations were determined. Thus, targeted Sanger sequencing beyond the *emb*B 306 codon or WGS together with phenotypic DST should be employed to ensure reliable ethambutol drug susceptibility testing, as a basis for the rational design of multidrug-resistant tuberculosis regimens with or without ethambutol.

## Abstract (precis)

Phenotypic drug susceptibility testing (DST) for the two first-line tuberculosis drugs ethambutol and pyrazinamide is known to yield unreliable and inaccurate results. In this prospective study, we propose a diagnostic algorithm combining phenotypic DST with Sanger sequencing to inform clinical decision-making for drug-resistant Mycobacterium tuberculosis complex isolates.

## INTRODUCTION

Isoniazid (INH), rifampin (RMP), ethambutol (EMB), and pyrazinamide (PZA) are first-line drugs used to treat tuberculosis (TB) caused by the fully susceptible Mycobacterium tuberculosis complex (MTBC) (1). In contrast, multidrug-resistant TB (MDR-TB) caused by MTBC resistant to both RMP and INH requires prolonged treatment with less efficacious and more toxic second-line drugs. Drug susceptibility testing (DST) is crucial to detect resistance, as it informs the choice of effective treatment. While phenotypic DST (pDST) provides reliable and reproducible results for INH and RMP, it yields less accurate results for EMB and PZA (2). Potential explanations include an inaccurate current critical concentration splitting the upper end of the wild-type distribution as well as methodological variations within diagnostic laboratories (3).

In the context of MDR-TB, EMB and PZA are recommended as “add-on agents” regardless of pDST results (4). Side effects caused by PZA and EMB, such as nausea, vomiting, hepatoxicity, and optic neuropathy, often accrete the multiple side effects caused by second-line drugs (5, 6). If reliable and reproducible DST for EMB and PZA was available, unnecessary exposure and harmful side effects could be avoided. With PZA being an integral part of the World Health Organization (WHO) recommended short-course MDR regimen, the reliable detection of PZA resistance is important for countries considering a roll-out of this regimen (4).

Molecular-based methods detecting resistance-conferring mutations proffer alternatives to pDST for select drugs. EMB targets the mycobacterial cell wall by inhibiting the arabinosyl transferases encoded by the *embCAB* operon, an ∼10-kb region comprising the three adjacent genes *embC*, *embA*, and *embB* (7). Its effect is mainly exerted upon the polymerization steps in the biosynthesis of the arabinan component of cell wall arabinogalactan (8). The *pncA*-encoded nicotinamidase/pyrazinamidase converts the prodrug PZA to pyrazinoic acid, which disrupts membrane permeability and transport. In accordance with these mechanisms, resistance to EMB and PZA are mainly associated with mutations in the *embCAB* operon, notably codon 306 of *embB*, and the *pncA* gene (9–12).

The present study aimed to investigate an algorithm combining phenotypic and genotypic (*pncA* and *embB*306, Sanger sequencing) approaches to determine PZA and EMB susceptibility in RMP-resistant TB isolates for routine use in a laboratory.

## RESULTS

A total of 85 unrelated clinical isolates, including 7 RMP-monoresistant and 78 MDR isolates were included. These isolates comprised all four main lineages of M. tuberculosis. A total of 43 isolates belonged to lineage 2 “Beijing” (50.6%), 10 to lineage 3 “Delhi-CAS” (11.8%), 2 to lineage 1 “East-African-Indian” (2.4%), and 30 to lineage 4 “Euro-American” (35.3%) (see Fig. S1 in the supplemental material). For a detailed list of all detected mutations known to be implicated in EMB and PZA resistance as well as the excluded phylogenetic single nucleotide polymorphisms (SNPs), see Table S1 in the supplemental material.

### Sequencing and phenotypic drug susceptibility.

pDST classified 52 isolates (61.2%) as PZA resistant. There was 100% concordance between pDST and *pnc*A sequencing results for 49 PZA-resistant and 33 PZA-susceptible isolates. No PCR results could be obtained for three resistant isolates. Next-generation sequencing (NGS) revealed large *pncA* deletions for two isolates and a *pncA* T47A mutation for one of these isolates. A total of 28 different mutations, including one double mutation, were identified in the *pncA* gene. *pncA* D8G, L4S, and T76P represented the most common variants (Table S1).

For EMB, 42 (49.4%) isolates tested resistant and 43 (50.6%) susceptible by pDST. Overall, 60 of the 85 isolates (70.6%) showed concordance between pDST and *embB*306 sequencing.

### Ethambutol-resistant isolates.

In less than two-thirds of the 42 EMB-resistant isolates (24 of 42, 57.14%), phenotypic resistance was confirmed by identifying a mutation of the *embB* codon 306 by Sanger sequencing. The remaining 18 isolates were phenotypically resistant to EMB without evidence of *embB*306 mutations. We repeated pDST for isolates with discordant results. Of 18 phenotypically resistant isolates with *embB*306 wild type, one isolate tested susceptible on repeat testing. For 16 of the remaining 17 strains, NGS analysis revealed nonsynonymous, nonphylogenetic mutations in the *embCAB* operon up- or downstream from the *emb*B306 codon. The mutation *embB* Q497R was present in 7 isolates (once in combination with *embB* A453A), and *embB* G406A was found in 5 isolates (once in combination with *embB* D1024N). The following SNPs were identified in only one isolate each: *embB* S297A together with *embB* D1024N, *embB* D354A with *embB* D1024N, and *embB* Q497K. The mutations *embB*Y319C and *embB* S297A were present only in pDST-resistant strains. Phenotypic resistance to EMB occurred at a significantly higher frequency in isolates that carried mutations at *embB* codon 306 or 497 (chi-square, *P* < 0.0001).

### Ethambutol-susceptible isolates.

**FIG 1:**
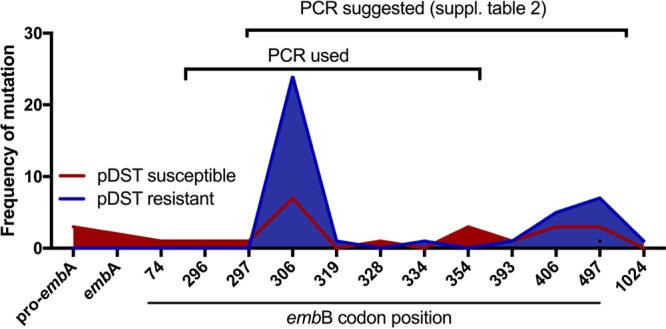
Distribution of mutations across the *embB* codons. Frequencies of mutations at their respective codon positions are shown. The region covered by the PCR primers currently used and the validated PCR primers suggested to be used (Table S2) are indicated by black brackets.

In total, 43 out of 85 isolates (50.6%) tested phenotypically susceptible. Among these isolates, 7 (16.3%) displayed a codon 306 (M306I) mutation in the *embB* gene, while the majority were found to be *embB*306 wild type (36 out of 43 susceptible isolates [83.7%]). Of these 7 phenotypically susceptible isolates with an *embB*306 mutation, 2 isolates tested resistant in a repeated pDST. Of the remaining 5 phenotypically susceptible isolates with an *embB*306 mutation, 4 displayed elevated MICs (Table 1). Aside from variants in the *embB*306 codon, 18 susceptible isolates had at least one mutation within *embCAB*. The mutations *embB*D354A, *embB*G406A, and *embB*Q497K occurred in both pDST-resistant and -susceptible isolates (Table 1; Fig. 1). Increased MICs were detected in 4 of the 5 susceptible isolates with these mutations. One isolate with an *emb*BD354A and an additional *embB*D1024N mutation was tested resistant when repeated. Of the remaining 31 susceptible isolates, with and without the *embCAB* mutation, 4 were tested resistant when repeated and 3 had a double mutation in *embB* (2 times Y334H + *embA* -12c/T and once *embB* D328G + *embB* L74R).

**TABLE 1:** *embCAB* mutations and their corresponding MICs in the selected clinical isolates

	pDST	
Mutation(s)	Susceptible (*n* = 43) (MICs, μg/ml^*a*^)	Resistant (*n* = 42)
*embA* -12c/t	2 (2.5, 3.75)	
*embA* -11c/a	1 (2.5)	
*embA* P838L	1 (≤1.25)	
*embB* N296H	1 (3.75)	
*embB* S297A + *embB* D1024N		1
*embB* M306I	6 (3.75 [*n* = 3], 5 [*n* = 1], ≥5^*b*^ [*n* = 2])	3
*embB* M306I + *embB* N296H		1
*embB* M306I + *embA* -16c/a		1
*embB* M306V	1 (2.5)	17
*embB* M306V + Q497P		1
*embB* M306V + *embA* -11c/t		1
*embB* Y319C		1
*embB* D328G + *embB* L74R	1 (≥5^*b*^)	
*embB* Y334H + *embA* -12c/t	2 (≥5^*b*^, ≥5^*b*^)	
*embB* D354A	1 (5)	
*embB* D354A + *embB* D1024N	1 (≥5^*b*^)	1
*embB* T393A	1 (≤1.25)	
*embB* G406A	1 (5)	4
*embB* G406A + *embB* D1024N		1
*embB* G406D	2 (≤1.25, 3.75)	
*embB* Q497K	2 (5, 5)	1
*embB* Q497R		4
*embB* Q497R + *embB* Q453A		1
*embB* Q497R + *embC* A387V		1
*embB* Q497R + *embA* -8c/a		1
*embB* D1024N	1 (3.75)	
*embC* P707L	1 (2.5)	
No mutation	18 (^*c*^)	2^*d*^

a MICs were tested for susceptible strains only.

b Tested resistant when repeated.

c ≤1.25 (*n* = 11),  2.5 (*n* = 6), ≥5 (*n* = 1) (gspI -240 c/T and Rv3785 c/G at position 4,243,217).

d One was tested susceptible, when repeated (MIC, ≤1.25).

No *embR* mutation was detected in our data set, while variants in *ubiA* and *aftA* were each found in three cases representing both susceptible and resistant isolates. The two EMB-resistant isolates with a variant in *ubiA* and one EMB-resistant isolate with a mutation in *aftA* also harbored mutations in the *embCAB* operon. All mutations found in EMB-resistant and -susceptible isolates within the *embCAB* operon and in other candidate genes and the MICs of EMB-susceptible strains are listed in Table S1 and Table 1.

## DISCUSSION

This study showed 100% concordance between pDST and *pncA* Sanger sequencing for PZA. However, our study affirms that pDST and *emb*B306 sequencing alone is not sufficient for a reliable determination of EMB resistance (13). Concordance with phenotypic resistance was high for some mutations identified within the *embCAB* operon, notably *embB* M306V and *embB*Q497R. Additional mutations that were present in both phenotypically susceptible and resistant isolates in our collection and were associated with elevated MICs comprise *embB*Q497K, *embB*D354A, and *embB*G406A. This is in agreement with previously published MIC data showing an association between these codons and high MICs (9, 14–16). The fact that some of these isolates were initially classified as susceptible by pDST is explained by isolate MICs close to the EMB critical concentration. Our data suggest that an isolate should be regarded as EMB resistant if any of these mutations are present and an elevated MIC is observed, regardless of the initial pDST result. Yet, because of the relatively small sample size, no conclusions can be drawn for other mutations detected in only one or two isolates.

Earlier work suggests that *ubiA* (Rv3806c) and notably nonsynonymous mutations in codons 237 and 240 may confer EMB resistance (17). We found three *ubiA* mutations in our data set (V55G and two times E149D) in both phenotypically resistant and susceptible isolates. *aftA*, another enzyme-encoding gene implicated in cell wall synthesis has also been found to be associated with EMB resistance (18). Our data set revealed mutations at codon 575 in a susceptible isolate and mutation T611M in a resistant and susceptible isolate.

pDST in combination with Sanger sequencing of codon 306 in *embB* correctly identified 57.1% (24/42) of EMB-resistant isolates. An additional 16 isolates would have been detected as EMB resistant if the region covered by Sanger sequencing was widened to include codons *embB*297 and *embB*497, as previously proposed (13, 19, 20). The sensitivity to predict phenotypic EMB resistance of the *embB* codon 306 mutations was 0.57 (specificity 0.83) which could be increased to 0.9 (specificity 0.67) when including *embB* codons 306, 354, 406, and 497. With all *embCAB* mutations identified among our study isolates, the sensitivity to predict EMB resistance was 0.95 (specificity 0.42). Primers for an extended PCR spanning these codons have been validated as part of this study (see Table S2 in the supplemental material). No PCR product could be obtained for three pDST-resistant isolates. The phenotypic resistance can be explained by larger deletions in two isolates and a T47A mutation in one isolate. The mutation T47A has been described to lead to increased MICs close to the resistance breakpoints (21).

In contrast to previous publications, we report 100% concordance between molecular and phenotypic PZA DST (22). NGS confirmed resistant pDST results in three additional isolates with noninterpretable Sanger sequencing results. Two of these isolates had large deletions in the *pncA* gene. A recent study involving six national reference laboratories investigating 1,142 MDR strains showed that 10% of isolates with *pncA* mutations tested phenotypically susceptible. The majority of *pncA* variants (85%) in this study were high-confidence mutations known to be associated with PZA resistance (11). In a comprehensive mutational screening approach, the PZA-resistance phenotype of 977 *pncA* nonsynonymous SNPs was assessed. One-third of the mutations (*n* = 301) were resistance conferring while another one-third (*n* = 310) were not associated with phenotypic resistance (23). Of the 28 different *pncA* mutations identified in our study, 18 belonged to the resistance-conferring group as per Yadon et al. (23); none were in the group not associated with resistance.

### Recommendation for patient management.

**FIG 2:**
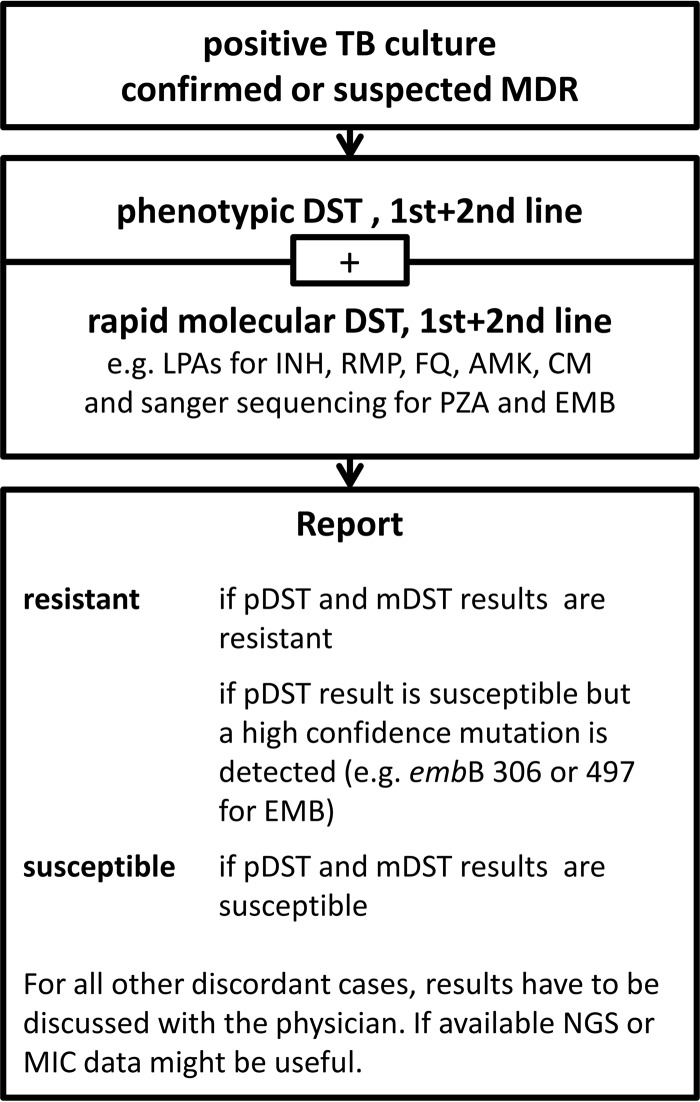
Algorithm proposed for ethambutol resistance determination in a mycobacterial laboratory in a low incidence-setting.

One method alone, i.e., pDST, Sanger sequencing around codon 306 of *emb*B, or NGS, is insufficient to reliably detect EMB resistance. Thus, we propose a diagnostic algorithm using phenotypic and genotypic methods in parallel for all clinical isolates with RMP resistance detected by rapid molecular (GeneXpert or line probe assays) or phenotypic tests (Fig. 2). If NGS is not available or only available as a research tool, Sanger sequencing between codon 297 and 497 of *embB* should be considered (see Table S2 for a validated primer sequence spanning these *embB* codons). Interpretation of DST results is straightforward if phenotypic and genotypic test results are in agreement. Discordances between phenotypic and genotypic results may need to be further investigated. However, isolates with mutations in the *embB* codons 306, 354, 406, and 497 should be assumed EMB resistant regardless of the phenotypic DST result (9, 14–16). MICs should be determined for all phenotypically susceptible isolates with mutations in the genes/operon *aftA*, *ubiA*, and *embCAB* to elucidate putative additive effects of linked mutations (e.g., *embB* S297A and *embB* D1024N) and the relevance of debatable mutations (e.g., *embB* Y319C and N296H) on the EMB-resistance level. If MIC testing is not feasible, repeated pDST at the critical concentrations should be performed. For those isolates, the microbiologist or biomedical scientist should relay the uncertainty about the effectiveness of EMB to the clinician who may or may not consider EMB as an add-on agent.

Implementation of the proposed algorithm is likely to be feasible and relevant in most low TB incidence, high-resource settings where pDST is routinely performed and access to NGS is widely available. The majority of MDR-TB cases in Western European countries are diagnosed among migrants from high TB and/or MDR-TB burden countries. In Germany, a high proportion of MDR-TB cases are from former Soviet Union and Eastern Europe countries (24). While the prevalence of EMB resistance among MDR-TB cases is not systematically reported, the studies reporting EMB resistance show a consistently high prevalence of >50% (25, 26).

The feasibility of implementing this algorithm in high MDR-TB burden middle or low-income settings depends on the availability of phenotypic DST and sequencing. This, in turn, requires technical skills and expertise, infrastructure, appropriate biosafety measures, and bioinformatics. Over the past years the capacity to perform phenotypic DSTs has greatly increased in many middle or low-income settings. There is great interest and enthusiasm to rolling out sequencing in low-resource settings, but wide implementation has not yet happened (27; https://unitaid.org/call-for-proposal/seeking-projects-to-fight-tuberculosis-and-its-drug-resistant-strains/#en). With sequencing becoming more widely available, algorithms such as the one proposed in this study will be implemented in high MDR-TB burden settings, where they are most needed.

### Conclusions.

At present, pDST for PZA and EMB cannot be replaced by any commercially available molecular diagnostic. The one line-probe assay aimed at detecting EMB resistance covers a limited range of resistance-conferring mutations and does not differentiate between resistance-conferring or silent variants. Thus, Sanger sequencing or NGS together with pDST should be employed to ensure reliable EMB DST results, enabling clinicians to decide whether to include EMB as part of an MDR-TB regimen.

## MATERIALS AND METHODS

### M. tuberculosis isolates.

We included all RMP-resistant clinical isolates referred to the German National Reference Laboratory for Mycobacteria, Borstel (NRL), between January 2016 and March 2017.

### Phenotypic and Sanger-based drug susceptibility testing.

*pncA* and *embB*306 Sanger sequencing and pDST for first and second-line drugs were done in parallel as part of the diagnostic service. pDST was performed using the MGIT960 system according to the manufacturer’s instructions (Becton, Dickinson, Sparks, MD). Processing of isolates and DNA extraction were performed as previously described (28). MGIT tubes were prepared with 0.8 ml MGIT960 SIRE supplement and, with exception of the drug-free growth control tubes, 0.1 ml drug solution. The following drug concentrations were tested: PZA at 100 and EMB at 5.0 μg/ml. Bacterial suspensions of 0.5 ml were added to the test tubes and the growth-control tubes.

For molecular DST, *pnc*A and *emb*B306 sequencing were performed as previously described (9, 29).

### MIC testing for ethambutol.

For EMB MIC determination, pDST-susceptible strains were subsequently tested at 5.0, 3.75, 2.5, and 1.25 μg/ml. The provided drug of the Bactec MGIT 960 SIRE kit was reconstituted into sterile distilled/deionized water as described in the package insert. For the test concentrations 3.75, 2.5, and 1.25 μl/ml, the stock solution was diluted 3:1, 1:1, and 1:3 with sterile distilled/deionized water before 100 μl were added to the respective MGIT tubes. The interpretation was done with the EpiCenter TBeXiST software according to Springer et al. (30).

### Next-generation sequencing and phylogenomic analyses.

All isolates underwent next-generation sequencing (NGS) to confirm mutations and investigate relatedness of isolates. From extracted genomic DNA, sequencing libraries were constructed using the Nextera XT kit and run on the NextSeq (2 × 150 bp) sequencing platform (Illumina, San Diego, CA, USA). Reads were mapped to the reference genome M. tuberculosis H37Rv (GenBank accession number NC_000962.3) with the alignment program BWA. Reads were refined using the Genome Analysis Toolkit (GATK) and SAMtools. For variant calling in the mapped reads, we used SAMtools and custom perl scripts with minimum thresholds of four reads in both forward and reverse orientation and 75% allele frequency. Variants in repetitive regions or genes were masked. Single nucleotide polymorphism (SNP) positions with a clear base call in all isolates were concatenated to a sequence alignment. A maximum likelihood tree was inferred using FastTree with 1,000 resamples (31). The consensus tree was midpoint rooted in FigTree, and nodes were arranged in increasing order.

### Accession number(s).

The sequence read sets were deposited in the European Nucleotide Archive under the BioProject accession number PRJEB27354.

## Supplementary Material

Supplemental file 1

Supplemental file 2
