# Supplementary material for: A Diagnostic Algorithm To Investigate Pyrazinamide and Ethambutol Resistance in Rifampin-Resistant Mycobacterium tuberculosis Isolates in a Low-Incidence Setting
Source: Antimicrob Agents Chemother. 2019 Jan 29;63(2):e01798-18. doi: 10.1128/AAC.01798-18 (PMC6355586; doi:10.1128/AAC.01798-18)
Supplement: Supplemental file 2 [file d56d112737a937d5acbc7ab6b0c81270_AAC.01798-18-s0002.pdf]

Supplemental Table 1: Molecular and phenotypic drug susceptibility data, accession numbers and genotype information for all isolates included in this study.

Supplemental Figure 1: Phylogenetic distribution of included isolates. Circular phylogramm of a maximum likelihood analysis inferred from 9472 SNPs positions shows that all major lineages are represented in our study sample. L = lineage; EAI = East African Indian

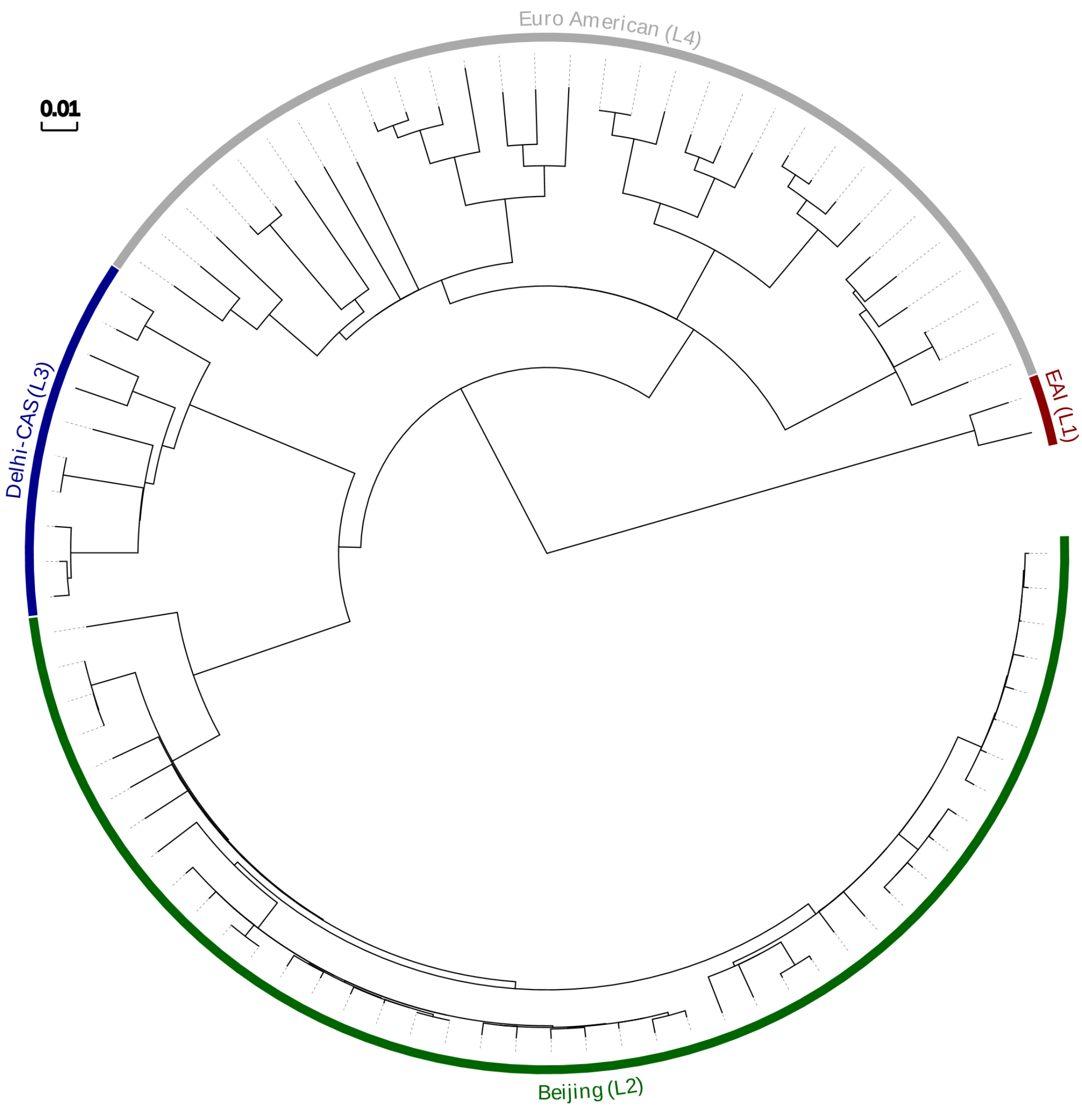

Supplemental table 2: (A) PCR-primer sequences used for Sanger sequencing around the *emB*306 codon and (B) PCR-primer sequences suggested tob e used to amplify an extended region oft he *embB* gene

A. PCR used in routine diagnostics to amplify the respective *embB* region

**Target**	**Primer**	**Sequence (5'-3')**	**Nucleotide Positions**	**Codon positions**	**Size (bp)**
embB	OG240 (embF545)	CgTTCCggCCTgCAT	764-778	255	344
	OG243 (embR887)	ACCTCACgCgACAgCA	1106-1091	369	

B. Validated PCR primers suggested to use for diagnostics to amplify the extended *em*bB region

**Target**	**Primer**	**Sequence (5'-3')**	**Nucleotide Positions**	**Codon positions**	**Size (bp)**
embB	Emb297-497F	TATTCggCTTCCTgCTCTgg	851-870	284	797
	Emb297-497R	gCATAgCgCggTgATCAAAA	1647-1628	549	
